# Editorial: Plant-based bioactive compounds: natural tumor prevention?

**DOI:** 10.3389/fnut.2023.1291615

**Published:** 2023-10-27

**Authors:** Hui Jia, Mei Feng

**Affiliations:** ^1^School of Traditional Chinese Medicine, Shenyang Medical College, Shenyang, China; ^2^Science and Experimental Research Center of Shenyang Medical College, Shenyang, China

**Keywords:** γ-secretase/Notch pathway, tumor resistance, natural γ-secretase inhibitors, cancer stem cells, EMT

Tumor drug resistance is an important clinical issue in the field of tumor therapy, it has been shown that the activation of γ-secretase/Notch signaling pathway can promote tumor drug resistance. The activation of the Notch pathway results in enhancement in small numbers of drug-resistant cells and stem-cell-like features in tumor, promotion of epithelial mesenchymal transition (EMT) to facilitate the transformation of tumor cells into cancer stem cells (CSCs) and an increasement in the expression of ATP-binding cassette transporter (ABC), all of which contribute to tumor drug resistance. The γ-secretase inhibitors (GSIs) inhibit γ-secretase/Notch pathway and Notch associated processes of CSCs repair and regeneration, EMT and tumor microenvironment (TME), and pro-tumor cell proliferation, thereby reversing tumor drug resistance. GSIs, as a new strategy to reverse tumor drug resistance, can directly reverse the resistance of tumor cells to traditional therapeutic agents or increase chemotherapy sensitivity. In addition, GSIs can induce the pro-apoptosis in tumor cells, thereby synergistically killing tumor cells with conventional chemotherapy drugs.

However, in clinical application to treat cancer chemotherapy resistance, GSIs have not achieved the expected results in reversing cancer chemoresistance. The exact mechanism is not yet understood, which may be related to the following factors: first, GSIs cannot selectively inhibit specific subtype of Notch receptor, while some subtypes of Notch receptor, such as Notch receptor 2, may be endowed with anti-tumor effects (1, 2); second, the inhibition of the Notch pathway may trigger protective autophagy in tumor cells, leading to tumor drug resistance; finally, GSIs may increase KLF4 expression, which promotes the accumulation of cupped cells in the intestine and gastrointestinal toxicity (3). Although there are still some controversies over reversal of drug resistant by GSIs, vast majority of studies support that GSIs can effectively reverse tumor drug resistance. Currently, it has been proposed following solutions to address the challenges associated with above-mentioned GSIs treatment of tumor drug resistance: First, improvement of specific inhibition of Notch receptors by optimization of GSIs design strategies, such as optimizing drug sequence length, altering target site selection. Second, reducing gastrointestinal toxicity by using intermittent doses of GSIs, or packaging GSIs into nanoparticles (4, 5). Finally, reversal of GSIs treatment resistance by adding PKC inhibitor into GSIs regimen (6).

The development of resistance to chemotherapy in cancer is a huge obstacle. In preclinical animal studies, drug activity is easily overestimated when treated alone, and combination therapy is an improvement on the above approach. During the implementation of combination therapy, patients need to be classified using biomarkers that can determine γ-secretase activity, thus selecting which patients are suitable for GSIs combined with conventional therapy. Therefore, the search for genes or markers that can reflect the activity of γ-secretase is key for combination therapy. In addition, phenotypic screening of tumors using patient samples can provide useful information to guide the development of next-generation GSIs and also for the evaluation of tumor cell targeted natural ingredients. Our research team confirmed that Cimigenoside, isolated and purified from Cimicifuga dahurica (Turcz.) Maxim, can target the inhibition of γ-secretase/Notch pathway, and show the activity of reversing paclitaxel resistance in triple negative breast cancer (7). Although the development of GSIs from natural active ingredients for reversal of chemoresistance holds some promise, there are still many challenges in bringing such novel GSIs from bench to bed.

## Author contributions

HJ: Writing—review & editing. MF: Writing—original draft.

## References

[1] Brzozowa-Zasada M Piecuch A Michalski M Segiet O Kurek J Harabin-Słowińska M. Notch and its oncogenic activity in human malignancies. Eur Surg. (2017) 49:199–209. 10.1007/s10353-017-0491-z 29104587 PMC5653712

[2] Chimento A D'Amico M Pezzi V De Amicis F. Notch signaling in breast tumor microenvironment as mediator of drug resistance. Int J Mol Sci. (2022) 23:6296. 10.3390/ijms23116296 35682974 PMC9181656

[3] Real PJ Tosello V Palomero T Castillo M Hernando E de Stanchina E. Gamma-secretase inhibitors reverse glucocorticoid resistance in T cell acute lymphoblastic leukemia. Nat Med. (2009) 15:50–8. 10.1038/nm.1900 19098907 PMC2692090

[4] Wang L Zi H Luo Y Liu T Zheng H Xie C. Inhibition of Notch pathway enhances the anti-tumor effect of docetaxel in prostate cancer stem-like cells. Stem Cell Res Ther. (2020) 11:258. 10.1186/s13287-020-01773-w 32586404 PMC7318403

[5] Zhou Y Guan L Li W Jia R Jia L Zhang Y. DT7 peptide-modified lecithin nanoparticles co-loaded with γ-secretase inhibitor and dexamethasone efficiently inhibit T-cell acute lymphoblastic leukemia and reduce gastrointestinal toxicity. Cancer Lett. (2022) 533:215608. 10.1016/j.canlet.2022.215608 35240234

[6] Franciosa G Smits JGA Minuzzo S Martinez-Val A Indraccolo S Olsen JV. Proteomics of resistance to Notch1 inhibition in acute lymphoblastic leukemia reveals targetable kinase signatures. Nat Commun. (2021) 12:2507. 10.1038/s41467-021-22787-9 33947863 PMC8097059

[7] Jia H Liu M Wang X Jiang Q Wang S Santhanam RK. Cimigenoside functions as a novel γ-secretase inhibitor and inhibits the proliferation or metastasis of human breast cancer cells by γ-secretase/Notch axis. Pharmacol Res. (2021) 169:105686. 10.1016/j.phrs.2021.105686 34022397

